# Antibiotic exposures and the development of pediatric autoimmune diseases: a register-based case–control study

**DOI:** 10.1038/s41390-022-02188-4

**Published:** 2022-07-19

**Authors:** Laura K. Räisänen, Sohvi E. Kääriäinen, Reijo Sund, Elina Engberg, Heli T. Viljakainen, Kaija-Leena Kolho

**Affiliations:** 1 Faculty of Medicine and Health Technology (MET), Tampere University, Tampere, Finland; 2 Folkhälsan Research Center, Helsinki, Finland; 3 Department of Pediatrics, Tampere University Hospital, Tampere, Finland; 4 Finnish Institute for Health and Welfare, Helsinki, Finland; 5 Institute of Clinical Medicine, School of Medicine, Faculty of Health Sciences, University of Eastern Finland, Kuopio, Finland; 6 Faculty of Medicine, University of Helsinki, Helsinki, Finland; 7 Children’s Hospital, Helsinki University Hospital, Helsinki, Finland

## Abstract

**Background:**

Antibiotics have been associated with several individual autoimmune diseases (ADs). This study aims to discover whether pre-diagnostic antibiotics are associated with the onset of ADs in general.

**Methods:**

From a cohort of 11,407 children, 242 developed ADs (type 1 diabetes, autoimmune thyroiditis, juvenile idiopathic arthritis (JIA), or inflammatory bowel diseases) by a median age of 16 years. Antibiotic purchases from birth until the date of diagnosis (or respective date in the matched controls *n* = 708) were traced from national registers.

**Results:**

Total number of antibiotic purchases was not related to the onset of ADs when studied as a group. Of specific diagnoses, JIA was associated with the total number of antibiotics throughout the childhood and with broad-spectrum antibiotics before the age of 3 years. Intriguingly, recent and frequent antibiotic use (within 2 years before diagnosis and ≥3 purchases) was associated with the onset of ADs (OR 1.72, 95% CI 1.08–2.74). Regardless of frequent use in childhood (40% of all antibiotics), penicillin group antibiotics were not related to any ADs.

**Conclusions:**

Use of antibiotics was relatively safe regarding the overall development of ADs. However, broad-spectrum antibiotics should be used considerately as they may associate with an increased likelihood of JIA.

**Impact:**

- Increasing numbers of antibiotic purchases before the age of 3 years or throughout childhood were not associated with the development of pediatric autoimmune diseases.
- Broad-spectrum antibiotics were related to the development of autoimmune diseases, especially juvenile idiopathic arthritis in children, while penicillin group antibiotics were not.
- The use of broad-spectrum antibiotics in children should be cautious as they may carry along a risk for autoimmune disease development.

## Introduction

Autoimmune diseases (ADs) are disorders in which the immune system attacks healthy tissues. Some ADs such as type 1 diabetes (DM), autoimmune thyroiditis (AIT), juvenile idiopathic arthritis (JIA), and inflammatory bowel diseases (IBD) may have overlapping genetic pathways^1,2^ and similarities in their pathogenesis involving T-cell organ inflitrations.^3–7^ The gut is the largest organ harboring T cells, and therefore is the place where most antigen-immune system contact occurs.^8^ To interact with the immune system, antigens have to penetrate the gut mucosal barrier and be introduced by the antigen-presenting cells to the T cells.^9^ Gut mucosal barrier may be breached due to disrupted gut microbiota homeostasis—for instance after antibiotic exposures, leading to influx of antigens and excess stimulation of the immune system.^10^ Sequentially, this may contribute to the onset of ADs.^11–14^

For unknown reasons, the incidences of pediatric-onset DM, AIT, JIA, and IBD in Finland are particularly high, presuming a presence of mutual environmental risk factors in addition to potential genetic predisposition.^15–19^ In the eastern neighboring countries of Finland (Estonia and Russian Karelia), the incidence of ADs such as DM is lower than in Finland. This finding was suggested to derive from different exposures to environmental pathogens and microbes.^20^ In line with this, Finnish children used more antibiotics during the first and second years of life compared with Estonian children, and this is reflected in the composition of the gut microbiota and immune stimulation.^21^ Previous studies have connected antibiotic exposures (especially in early childhood) with the onset of JIA and Crohn’s disease, while findings regarding DM were controversial.^22–27^ AIT has been related to tetracycline use in adolescence, but the mechanism has remained unspecified.^28^

Most of the previous studies on exposures to antibiotics and the development of ADs addressed each disease individually in different settings, making it challenging to estimate whether antibiotic exposures could be associated with the development of pediatric autoimmunity in general, yet manifesting as different diseases. This register-based case–control study focuses on the potential relationship between antibiotics and the onset of ADs in general.^22–25^ Our aim was to investigate whether the development of ADs (represented by DM, AIT, JIA, and IBD) is associated with (1) number of antibiotic exposures during different periods in childhood and (2) exposures to particular types of antibiotics.

## Methods

### Data sources for the study population

The study population was derived from Finnish Health in Teens (Fin-HIT) cohort—a nationwide prospective school-based cohort to address health behaviors of Finnish children and adolescents, comprising 11,407 children (born 2000–2005) without specific exclusion criteria. More details on recruitment and characteristics of the cohort have been described elsewhere.^29^ The cohort represents children from densely populated areas across Finland, with relatively high maternal socioeconomic status.^15^ Using a unique personal identity code of every Finnish resident, children in the Fin-HIT cohort were linked to three well-established national registers: (1) the Special Reimbursement Register (SRR)—containing records on patients with chronic diseases requiring medication (including entry dates and physician verified diagnosis), who are entitled to drug refunds regardless of their socioeconomic status; (2) the Drug Purchase Register (DPR)—containing data on all purchased drugs by prescriptions in Finland (including dispensation dates and pharmaceutical information); and (3) the Medical Birth Register (MBR)—containing information on gestational age, delivery modes, and postnatal antibiotic treatment before discharge. These registers are maintained by the Finnish Social Insurance Institution (SRR and DPR) and Finnish Institute for Health and Welfare (MBR).

### Identifying children for the matched case–control study

The outcome of this study was the diagnosis of at least one AD by the end of the follow-up on December 31, 2018—when the median age of the participants was 16 years. DM, JIA, and IBD (including Crohn’s disease, ulcerative colitis (UC), and IBD unclassified (IBDU)) diagnoses were obtained from the SRR using International Classification of Diseases, 10th revision (ICD-10) codes: E10 for DM; M08 for JIA; K50 (Crohn’s disease) and K51 (UC/IBDU) for IBD. AIT diagnoses were obtained from the DPR using ATC (Anatomical Therapeutic Chemical) code H03AA01 for thyroxin—the prescription-only drug used for AIT. The excellent coverage of these registers has been described previously.^30,31^ DPR was chosen for identifying AIT because thyroxin is inexpensive and therefore, not everyone using this medication is applying special reimbursement and registered in the SRR.

Of the 11,407 children in the Fin-HIT cohort, 242 developed a primary AD after the first year of life and generated the case group. Depending on the availability of potential controls, each child in the case group was matched with one to four children from the same cohort of similar age (0–4 days of differences in age to ensure an equal length of the observation period for potential antibiotic exposures), sex, and residential area (to decrease the impact of other environmental factors). Furthermore, preterm birth has been identified as a potential risk factor for ADs.^15^ Therefore, gestational age (preterm/term), and delivery mode (cesarean section/vaginal delivery) were also considered in the matching. Due to the limited number of potential controls, most children born preterm and/or with cesarean section had only one matching control.

### Number and types of antibiotic purchases

Data on perinatal antibiotic treatment during pregnancy and in the birth hospital were obtained from the DPR and MBR, respectively. Outpatient antibiotic purchases were collected from the DPR using ATC codes starting with J01. The data were collected from birth until two months prior to the index date (date of diagnosis for children with ADs/compatible date for their matched controls). The two-month period was chosen to reduce the possibility of including antibiotic purchases during the symptomatic phase of ADs.

**Fig. 1:**
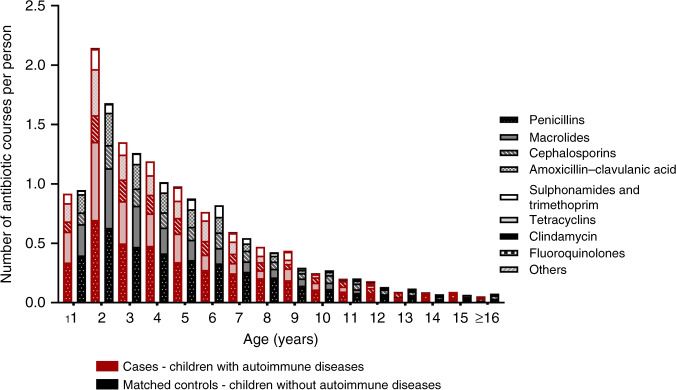
Purchased antibiotic courses before index date^a^ in the matched case–control^b^ study. The numbers of tetracycline, clindamycin, fluoroquinolone, and other antibiotics courses were relatively low. ^a^Index date = date of diagnosis for children who developed autoimmune diseases (type 1 diabetes, autoimmune thyroiditis, juvenile idiopathic arthritis, and inflammatory bowel diseases) and respective date for their matching controls. ^b^Cases = 242 children who developed autoimmune diseases by the end of follow-up (December 31, 2018) at a median age of 16 years. Each child in the case group was matched with one to four children of similar age, sex, residential area, gestational age (preterm/term), and delivery method (cesarean section/vaginal), comprising control group of 708 children.

**Fig. 2:**
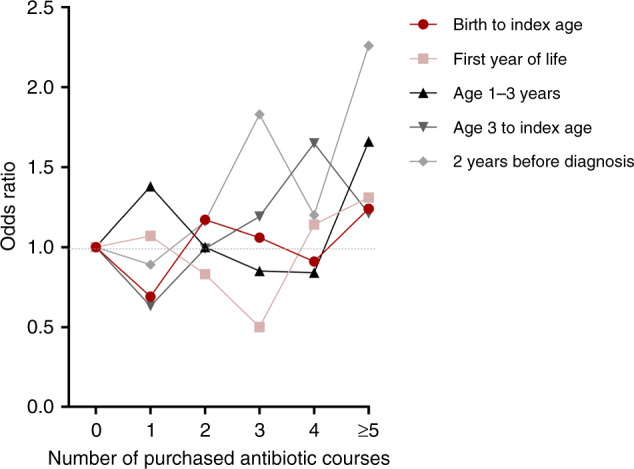
Nonlinear relationship between the number of antibiotic purchases in different periods of childhood and onset of autoimmune diseases^a^. ^a^Autoimmune diseases in this study were type 1 diabetes, autoimmune thyroiditis, juvenile idiopathic arthritis, and inflammatory bowel diseases. Index date = date of diagnosis for children who developed autoimmune diseases and compatible date for their age, sex, residential areas, gestational age (preterm/term), and delivery method (Cesarean section/vaginal delivery) matched controls.

Antibiotic exposures were analyzed in several observation periods based on the age distribution of antibiotic purchases (Fig. 1): (1) throughout childhood—from birth to the index date; (2) during the first year of life (infancy); (3) in the toddler phase—from the age 1 year up to third birthday/index date; (4) during preschool to adolescence—from the age of 3 years to the index date; and (5) within 2 years before the index date. The association between pre-diagnostic antibiotic purchases in each observation period and the development ADs as a group or individually as diagnoses of DM, AIT, JIA, or IBD were analyzed. Due to observed nonlinear associations between antibiotic exposures and the onset of ADs (Fig. 2), the antibiotic purchases were categorized into different groups. When concerning the total number of antibiotic exposures until the index date we used three groups: <4 courses; 4–8 courses; and >8 courses. Regarding shorter observation periods antibiotic purchases were categorized into three groups as follows: no purchases; 1–2 courses (occasional); and ≥3 courses (frequent).

Different types of antibiotics were categorized based on ATC codes (Supplementary Table 2) into five groups: (A) penicillins; (B) macrolides; (C) cephalosporins; (D) amoxicillin-clavulanic acid; and (E) sulfonamides and trimethoprim. Clindamycin, tetracyclines, fluoroquinolones, and other antibiotics such as nitrofurantoin and metronidazole were considered in the analysis regarding the total number of antibiotic purchases but dismissed from the subgroup analysis due to low frequency of usage.

### Statistical analysis

The background data of cases and controls are presented as mean and standard deviation (SD), median (interquartile range, IQR), or number/proportion (%). A matched case–control study design with an equal length of the observation period in which these exposures may occur in cases and in their controls was used. The pre-diagnostic antibiotic exposures of each case were compared with the antibiotic exposures of his/her matched control(s) until the index date, and the association between antibiotic exposures and the development of ADs was estimated using conditional logistic regression with strata analysis.^32^ Results were presented with odds ratio (OR) and 95% confidence interval (CI). The software used was IBM SPSS Statistics 26.0 and a 5% statistical significance level was adopted.

## Results

The background characteristics of the 242 children who developed ADs (cases) and their 708 matched controls who did not develop ADs are presented in Table 1. The ADs were diagnosed at a median age of 11 (IQR 6–13.8) years. The distribution of antibiotic purchases by age for cases with AD and their controls are shown in Fig. 1. A similar age-related pattern in purchases was noted when cases and controls were compared. Of all purchased antibiotics, 44.3% (*n* = 3825) were bought before the age of 3 years (Fig. 1). Throughout childhood, only 14 (5.8%) children in the case group and 34 (4.8%) in the matched control group had no antibiotic purchases (*p* = 0.596). Age at and the type of the first antibiotic purchase did not differ between cases and matched controls (Supplementary Table 3). Also, perinatal antibiotic exposures were similar between cases and their controls (Table 1).

**Table 1:** Background characteristics of children in the study population.

	DM (*N* = 102)	AIT (*N* = 68)	JIA (*N* = 54)	IBD (*N* = 27)	Cases^a^ (*N* = 242)	Matched controls^b^ (*N* = 708)
Age at the end of follow-up (years), mean ± SD	16.5 ± 1.6	17.1 ± 1.1	16.6 ± 1.3	16.8 ± 1.2	16.7 ± 1.4	16.8 ± 1.4
Sex, *N* (%)						
Girl	41 (40.2)	47 (69.1)	43 (79.6)	13 (48.1)	140 (57.9)	407 (57.5)
­ Boy	61 (59.8)	21 (30.9)	11 (20.4)	14 (51.9)	102 (42.1)	301 (42.5)
Residential area, *N* (%)						
­ Capital (south)	34 (33.3)	20 (29.4)	10 (18.5)	11 (40.7)	72 (29.8)	248 (35.0)
Inner south	6 (5.9)	7 (10.3)	13 (24.1)	4 (14.8)	30 (12.4)	93 (13.1)
West	9 (8.8)	18 (26.5)	10 (18.5)	2 (7.4)	36 (14.9)	92 (13.0)
East	33 (32.4)	18 (26.5)	9 (16.7)	5 (18.5)	63 (26.0)	161 (22.7)
North	20 (19.6)	5 (7.4)	12 (22.2)	5 (18.5)	41 (16.9)	114 (16.1)
Age of diagnosis/age at the index date, *N* (%)						
­ <6 years	36 (35.3)	3 (4.4)	19 (35.2)	2 (7.4)	59 (24.4)	154 (21.8)
6–12 years	42 (41.2)	21 (30.9)	18 (33.3)	11 (40.7)	88 (36.4)	273 (38.6)
>12 years	24 (23.5)	44 (64.7)	17 (31.5)	14 (51.9)	95 (39.3)	281 (39.7)
Gestational age, *N* (%)						
­ Term (≥37 weeks)	90 (88.2)	63 (92.6)	49 (90.7)	24 (88.9)	217 (89.7)	682 (96.3)
Preterm (<37 weeks)	9 (8.9)	4 (5.9)	4 (7.4)	3 (11.1)	20 (8.3)	26 (3.7)
Missing	3 (2.9)	1 (1.5)	1 (1.9)	0	5 (2.0)	0
Delivery mode, *N* (%)						
Vaginal	80 (78.4)	58 (85.3)	47 (87.0)	21 (77.8)	198 (81.8)	644 (91.0)
Cesarean section	19 (18.6)	9 (13.2)	7 (13.0)	6 (22.2)	40 (16.5)	64 (9.0)
Missing	3 (2.9)	1 (1.5)	0	0	4 (1.7)	0
Maternal antibiotic purchase during pregnancy, *N* (%)						
­ None	79 (77.5)	58 (85.3)	45 (83.3)	23 (85.2)	198 (81.8)	776 (80.9)
Up to 60 days before delivery	5 (4.9)	1 (1.5)	0	0	6 (2.5)	36 (3.8)
>60 days before delivery	18 (17.6)	9 (13.2)	9 (16.7)	4 (14.8)	38 (15.7)	147 (15.3)
Postnatal antibiotic exposure at the birth hospital before discharge						
None	96 (94.1)	64 (94.1)	49 (90.7)	24 (88.9)	224 (92.6)	679 (95.9)
Received antibiotics	5 (4.9)	3 (4.4)	5 (9.3)	3 (11.1)	16 (6.6)	29 (4.1)
Missing	1 (1.0)	1 (1.5)	0	0	2 (0.8)	0

^a^Cases = children with autoimmune diseases (represented with DM (type 1 diabetes mellitus), *AIT* (autoimmune thyroiditis), *JIA* (juvenile idiopathic arthritis), and *IBD* (inflammatory bowel diseases)). Nine children have two diagnoses.

^b^Each child in the case group was matched with one to four children of similar age, sex, residential area, gestational age (preterm/term), and delivery mode (cesarean section/vaginal). Due to limited potential controls, most children born preterm/with cesarean section have only one control instead of four.

### Number of antibiotic purchases

The total number of antibiotic purchases from birth to the index date was not associated with the development of the studied ADs as one group (Table 2). However, we found a nonlinear and timing-dependent relationship between the number of antibiotic exposures and the onset of ADs (Fig. 2). The highest odds for developing ADs were observed in children receiving ≥3 courses of antibiotics within 2 years before the index date when compared with those without antibiotic purchases (OR 1.72, 95% CI 1.08–2.74) (Table 2). The median age at this stage was 9 (IQR 4–12) years.

**Table 2:** Association between numbers of antibiotic purchases in different periods and the development of an autoimmune disease (DM, AIT, JIA, or IBD)^a^.

Antibiotic purchases at different ages, *N* (%)	Cases^a^ (*N* = 242)	Matched controls^b^ (*N* = 708)	Odds ratio (95% CI)^c^
*Throughout childhood (from birth to the index date)*^d^			
AD			
<4 courses	53 (21.9)	174 (24.6)	1.00 (Ref)
4–8 courses	82 (33.9)	223 (31.5)	1.32 (0.87–2.01)
>8 courses	107 (44.2)	311 (43.9)	1.22 (0.79–1.88)
DM			
<4 courses	28 (27.5)	81 (28.9)	1.00 (Ref)
4–8 courses	36 (35.3)	101 (36.1)	1.07 (0.60–1.93)
<8 courses	38 (37.3)	98 (35.0)	1.27 (0.69–2.36)
AIT			
<4 courses	11 (16.2)	25 (13.2)	1.00 (Ref)
4–8 courses	24 (35.3)	52 (27.4)	1.08 (0.45–2.58)
>8 courses	33 (48.5)	113 (59.5)	0.68 (0.29–1.60)
JIA			
<4 courses	10 (18.5)	56 (35.9)	1.00 (Ref)
4–8 courses	15 (27.8)	45 (28.8)	*2.91 (1.05–8.05)*
>8 courses	29 (53.7)	55 (35.3)	*6.60 (2.12–20.5)*
IBD			
<4 courses	5 (18.5)	11 (15.1)	1.00 (Ref)
4–8 courses	9 (33.3)	21 (28.8)	0.90 (0.21–3.86)
>8 courses	13 (48.1)	41 (56.2)	0.69 (0.17–2.79)
*Infancy (<age of 1 year)*			
AD			
None	128 (52.9)	356 (50.3)	1.00 (Ref)
1–2 courses	85 (35.1)	262 (37.0)	0.98 (0.71–1.36)
≥3 courses	29 (12.0)	90 (12.7)	0.86 (0.53–1.38)
DM			
None	59 (57.8)	147 (52.5)	1.00 (Ref)
1–2 courses	34 (33.3)	102 (36.4)	0.92 (0.55–1.53)
≥3 courses	9 (8.8)	31 (11.1)	0.58 (0.24–1.39)
AIT			
None	37 (54.4)	89 (46.8)	1.00 (Ref)
1–2 courses	22 (32.4)	73 (38.4)	0.73 (0.38–1.41)
≥3 courses	9 (13.2)	28 (14.7)	0.73 (0.31–1.74)
JIA			
None	24 (44.4)	82 (52.6)	1.00 (Ref)
1–2 courses	19 (35.2)	56 (35.9)	1.32 (0.65–2.68)
≥3 courses	11 (20.4)	18 (11.5)	2.26 (0.93–5.54)
IBD			
None	14 (51.9)	31 (42.5)	1.00 (Ref)
1–2 courses	12 (44.4)	29 (39.7)	0.99 (0.41–2.40)
≥3 courses	1 (3.7)	13 (17.8)	0.21 (0.03–1.75)
*Toddler phase (from age of 1 up to third birthday/the index date)*^d^			
AD			
None	45 (23.3)	146 (20.6)	1.00 (Ref)
1–2 courses	73 (30.2)	216 (30.5)	1.12 (0.71–1.76)
≥3 courses	124 (51.2)	346 (48.9)	1.16 (0.76–1.78)
DM			
None	21 (20.6)	56 (20.0)	1.00 (Ref)
1–2 courses	27 (26.5)	96 (34.3)	0.71 (0.36–1.43)
≥3 courses	54 (52.9)	128 (45.7)	1.17 (0.62–2.21)
AIT			
None	11 (16.2)	40 (21.1)	1.00 (Ref)
1–2 courses	25 (36.8)	44 (23.2)	2.48 (1.00–6.17)
≥3 courses	32 (26.4)	106 (55.8)	1.18 (0.50–2.81)
JIA			
None	13 (24.1)	35 (22.4)	1.00 (Ref)
1–2 courses	12 (22.2)	57 (36.5)	0.51 (0.18–1.46)
≥3 courses	29 (53.7)	64 (41.0)	1.44 (0.60–3.45)
IBD			
None	2 (7.4)	10 (13.7)	1.00 (Ref)
1–2 courses	9 (33.3)	17 (23.3)	6.85 (0.74–63.1)
≥3 courses	16 (59.3)	46 (63.0)	4.20 (0.49–35.8)
*Preschool to adolescence (from age of 3 years to index date*			
AD			
None	33 (13.6)	96 (13.6)	1.00 (Ref)
1–2 courses	38 (15.7)	147 (20.8)	0.82 (0.45–1.48)
≥3 courses	151 (62.3)	40 (57.3)	1.28 (0.75–2.18)
DM			
None	20 (19.6)	54 (19.3)	1.00 (Ref)
1–2 courses	13 (12.7)	71 (25.4)	0.59 (0.24–1.49)
≥3 courses	57 (55.9)	126 (45.0)	1.60 (0.73–3.53)
AIT			
None	7 (10.3)	13 (6.8)	1.00 (Ref)
1–2 courses	10 (14.7))	29 (15.3)	0.55 (0.17–1.85)
≥3 courses	51 (75.0)	148 (77.9)	0.64 (0.23–1.81)
JIA			
None	6 (11.1)	27 (17.3)	1.00 (Ref)
1–2 courses	9 (16.7)	35 (22.4)	1.89 (0.52–6.78)
≥3 courses	33 (61.1)	72 (46.2)	*3.94 (1.16*–*13.4)*
IBD			
None	1 (3.7)	2 (2.7)	1.00 (Ref)
1–2 courses	6 (16.2)	12 (16.4)	1.16 (0.75–17.8)
≥3 courses	18 (66.7)	52 (71.2)	0.80 (0.06–10.3)
*Purchases within 2 years before the index date*			
Autoimmune diseases			
None	120 (49.6)	371 (52.4)	1.00 (Ref)
1–2 courses	70 (28.9)	235 (33.2)	0.98 (0.69–1.40)
≥3 courses	52 (21.5)	102 (14.4)	*1.72 (1.08*–*2.74)*
DM			
None	54 (52.9)	153 (54.6)	1.00 (Ref)
1–2 courses	27 (26.5)	83 (29.6)	0.93 (0.53–1.63)
≥3 courses	21 (20.6)	44 (15.7)	1.34 (0.62–2.87)
AIT			
None	39 (57.4)	101 (53.2)	1.00 (Ref)
1–2 courses	21 (30.9)	64 (33.7)	1.11 (0.56–2.18)
≥3 courses	8 (11.8)	25 (13.2)	0.92 (0.33–2.55)
JIA			
None	24 (44.4)	76 (48.7)	1.00 (Ref)
1–2 courses	14 (25.9)	56 (35.9)	0.91 (0.39–2.10)
≥3 courses	16 (29.6)	24 (15.4)	2.39 (0.95–6.00)
IBD			
None	10 (37.0)	37 (50.7)	1.00 (Ref)
1–2 courses	9 (33.3)	27 (37.0)	1.25 (0.44–3.56)
≥3 courses	8 (29.6)	9 (12.3)	3.85 (0.93–15.9)

^a^AD = all autoimmune diseases together. Cases = children with ADs, *N* = 242 (represented with DM (type 1 diabetes mellitus), *N* = 102; *AIT* (autoimmune thyroiditis), *N* = 68; *JIA* (juvenile idiopathic arthritis), *N* = 54; and *IBD* (inflammatory bowel diseases), *N* = 27). Nine children had two diagnoses . Throughout childhood, only 48 children (5.8%) had no record of antibiotic purchases.

^b^Each child in the case group was matched with one to four children of similar age, sex, residential area, gestational age (preterm/term), and delivery mode (cesarean section/vaginal).

^c^Odds ratio and CI (95% confidence interval) were obtained using conditional logistic regression.

^d^Antibiotic purchases throughout childhood including postnatal antibiotics. Only 14 children in the case group and 34 in the control group had no antibiotic purchases.

Index date = date of diagnosis for children who developed autoimmune diseases and compatible date for their matching controls.

Italic values indicate statistical significance *p* < 0.05.

Regarding individual diagnoses, onset of JIA was more common in children receiving more than 4 courses of antibiotics (4–8 courses OR 2.91, 95% CI 1.05–8.05 and >8 courses OR 6.60, 95% CI 2.12–20.5) than in those receiving <4 antibiotic courses through the entire study period from birth to the index date (Table 2). Also, the development of JIA was associated with ≥3 antibiotic courses during preschool to adolescence when compared to the respective group with no antibiotic purchases (OR 3.94, 95% CI 1.16–13.4). No such associations were observed regarding DM, AIT, and IBD (Table 2).

### Types of antibiotic purchases

**Fig. 3:**
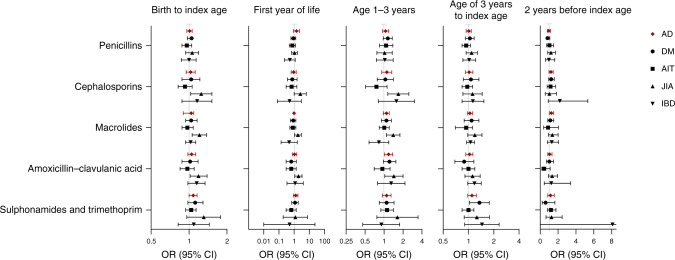
Association between types of antibiotic purchases in different exposure periods and the development of an autoimmune disease (AD), represented by type 1 diabetes (DM), autoimmune thyroiditis (AIT), juvenile idiopathic arthritis (JIA), or inflammatory bowel diseases (IBD)^a^. ^a^Cases = children with ADs (DM, AIT, JIA, or IBD). Nine children have two diagnoses. OR odds ratio, CI confidence interval. Analyses were performed using conditional logistic regression. Index date = age of diagnosis for children who developed autoimmune diseases and compatible date for their matching controls.

Penicillins were the most commonly purchased antibiotics (40% of all antibiotics, of which over 80% were amoxicillin), followed by macrolides (20% of all antibiotics, of which over 80% were azithromycin) (Supplementary Table 2). When purchases of penicillins, macrolides, cephalosporins, amoxicillin-clavulanic acid, sulfonamides, and trimethoprim were analyzed separately, none of them was associated with the onset of ADs in general (Fig. 3 and Supplementary Table 4). However, during the toddler phase purchases of amoxicillin-clavulanic acid (OR 1.18 95% 1.01–1.37); and within 2 years before the index date purchases of macrolides were associated with the onset of ADs in general (OR 1.24, 95% CI 1.01–1.51).

For individual diagnoses, the development of JIA was associated with purchases of broad-spectrum antibiotics (cephalosporins, macrolides, and amoxicillin-clavulanic acid), (Fig. 3 and Supplementary Table 4) in three different time periods: throughout childhood, infancy, and toddler phase. The highest ORs for these antibiotics were seen in infancy (cephalosporins OR 2.54, 95% CI 1.01–6.38; macrolides OR 1.80, 95% CI 1.08–3.01; and amoxicillin-clavulanic acid OR 1.93, 95% CI 1.12–3.32, respectively). On the other hand, the development of DM was associated with purchases of sulfonamides and trimethoprim during preschool to adolescence (OR 1.35, 95% CI 1.03–1.77) (Fig. 3 and Supplementary Table 4). These findings did not apply to any other individual diagnoses. Finally, purchases of penicillin were not associated with any types of ADs in this study.

## Discussion

Our study is the first to investigate the association of the number and types of antibiotic exposures in different stages of childhood with the onset of four common pediatric ADs (DM, AIT, JIA, or IBD) in a mutual setting. The total number of antibiotic purchases from birth to the index date was not associated with the development of the studied ADs as one group. However, we found a nonlinear and timing-dependent relationship between the number of antibiotic exposures and the onset of ADs. Furthermore, although the total exposure to antibiotics throughout childhood was not related to the development of these ADs, the more recent and frequent exposures within 2 years prior to the diagnosis were associated. This finding was further supported by purchases of macrolides 2 years prior diagnosis, which increased the risk of ADs. Regarding specific diagnosis of AD, the number of antibiotic exposures throughout childhood was associated with the onset of JIA. Intriguingly, early exposures to broad-spectrum antibiotics were associated with JIA as well. Despite being the most common antibiotic used in childhood, penicillins (predominantly amoxicillin) were safe to use at any age in relation to the development of ADs.

Antibiotic purchases during the first year of life were not associated with the development of any ADs. On contrary, higher exposures to antibiotics at later stages, i.e., close to the age of diagnosis (the median age of 9 years) were associated with the onset of ADs. How do these findings align with previous studies? A Swedish register-based study presented a connection between prescribed antibiotics during infancy and the onset of DM,^26^ while studies from other countries assessing parental reports or prescription records have not reported a significant association between early childhood antibiotic exposures and DM.^27,33,34^ Studies from the United Kingdom, Finland, and Sweden have associated early life antibiotic exposures with the onset of JIA.^22,23,35^ As for IBD, early antibiotic exposures have been related to Crohn’s disease, but this association was not apparent regarding UC.^25,36,37^ However, a recent meta-analysis did not confirm the relationship between antibiotics and IBD.^38^

Most studies have assumed a linear association between antibiotic exposure and the onset of a particular AD, i.e. the risk increases with increasing antibiotic exposure. Our study challenges this presumption, as we did not detect linear associations between antibiotic exposures and onset of ADs. In fact, penicillins (as the most common antibiotic type to treat pediatric infections) were not related to the development of any ADs at any age. Since infancy is the most susceptible period for common infectious diseases and consequently the period of most frequent antibiotic use,^39^ it is reassuring that antibiotics used in early childhood hardly increased the risk for developing pediatric ADs.

We reported that recent purchases of macrolides (within the two years before a diagnosis) were related to obtaining an AD in general, while purchases of sulfonamides and trimethoprim during preschool and adolescence were particularly associated with DM. Exposures to amoxicillin-clavulanic acid during the toddler phase were related to the development of an AD as well, but this finding was most likely driven by the association between this antibiotic and JIA. Intriguingly, in addition to amoxicillin-clavulanic acid, the use of other broad broad-spectrum antibiotics such as macrolides and cephalosporins before the age of three years was also associated with JIA but not with DM, AIT, or IBD. The reason for this finding can only be speculated. JIA is a group of complex, multifactorial, and heterogenous diseases.^40^ The pathogenesis of JIA involves several types of immunological cells, with interacting mechanisms that are not entirely known. For instance, JIA has been treated with non-steroid anti-inflammatory drugs, which have not been used in other ADs—suggesting a broader spectrum of inflammatory responses in its disease mechanism. Therefore, we suggest that early childhood infections, antibiotics, or both of them together, might influence a disease mechanism of JIA that is rather different than those of other ADs. What this disease mechanism might be is still beyond our understanding and warrants further studies.

Antibiotic exposures could be interpreted as exposures to infections, which might act as triggers for ADs.^41^ In our study, penicillins were the most common antibiotics used, yet having no prominent association with ADs. Furthermore, a recent cohort study from Sweden showed that while early antibiotic use was associated with JIA, the infections causing the antibiotic exposures were not.^35^ Finally, both antibiotic use (especially among children under the age of 5 years) and ADs are more common in industrialized countries than in developing countries, while infections are generally more common in developing countries.^42–46^ Therefore, infections may not be the most plausible explanation in relating antibiotics and ADs.

Since antibiotics have been shown to have an influence on gut microbial homeostasis,^47^ antibiotic exposures could be related to ADs through altering gut microbiota composition, often seen in different autoimmune diagnosis.^12,48–53^ Magnitude and type of gut microbiota modification varies according to given antibiotics, hence recovery time after different types of antibiotic exposures may vary as well.^54–56^ For example, macrolides targeting and inhibiting intracellular ribosomal protein synthesis have both a broad spectrum and a long-term influence on gut microbiota that may persist even for several years.^57–59^ In addition, macrolides have immunomodulatory properties.^60^ These characteristics may yield a summative response in the immune system. In our study, azithromycin was the most often used antibiotic among macrolides. Azithromycin has a broad bacteriostatic spectrum, a marked tissue penetration, a high stability, and a low clearance rate due to its long half-life, which enable it to reach a higher cellular concentration compared to penicillin.^57^ These characteristics may explain the long-term influences of azithromycin on gut microbiota compared with penicillin. Furthermore, a previous Fin-HIT study showed that azithromycin presented the strongest inverse association with salivary microbiota diversity.^61^ Since dysbiosis of gut and salivary microbiota have also been associated with ADs,^62^ we suggest that macrolides might catalyze long-term dysbiosis, explaining their association with ADs. Further studies to examine the potential link between the use of broad-spectrum antibiotics, the duration of their influence on gut microbiota, gut dysbiosis, and the onset of ADs are warranted.

The strength of our study lies in the comprehensive and excellent coverage of longitudinal data from national registers, which has been shown before.^63^ For example, we were able to trace purchased antibiotics as outpatients rather than just prescribed. In addition, we studied several ADs in a mutual setting, using a comprehensive Fin-HIT cohort with small variations in socioeconomic status as the source of the study population.^15^ The controls were matched for age (with a maximum difference of four days), sex, residential area, gestational age, and delivery method to limit the number of potential confounding factors. This matching design provided an additional benefit by indirectly limiting the role of the season as a confounder—since season-related factors, such as infections and daylight exposures, would similarly influence both cases and controls. In addition, our study setting made it possible to examine the association between childhood antibiotic exposures at different stages of childhood and onsets of the four pediatric ADs together, and to reliably compare one disease to another.

As for limitations, we lack information on the children’s genetic susceptibility to infections or to ADs. We also did not know why the antibiotics were purchased—for treating infections (and if so, for what kind of infection) or for prophylactic purposes—and on whether secondary antibiotic courses for the same infection were needed. In addition, we had no access to the antibiotics given during inpatient care. Yet, antibiotic treatments during hospitalization are often continued orally after discharge, and our data cover these post-discharge antibiotic purchases. Finally, we have no guarantee on the consumption of the purchased antibiotics. However, since monitoring antibiotic consumption of over 11,000 children for over a decade is technically not possible, a study design based on antibiotic purchases is the second-best option, which we used in this study.

## Conclusion

Use of antibiotics throughout childhood can be considered relatively safe in relation to the development of pediatric ADs. Antibiotics in the penicillin group are unlikely to be associated with the development of any ADs. In contrast, broad-spectrum antibiotics should be used considerately as they may associate with the development of ADs, especially JIA. In conclusion, the development of an AD is a multifactorial process in which antibiotics have a role to play, but the importance of that role still needs to be determined.

## Supplementary information

Supplementary Information

## Data Availability

The datasets generated during and/or analyzed in the current study are available from the corresponding author on reasonable request.
