# Supplementary material for: Antibiotic exposures and the development of pediatric autoimmune diseases: a register-based case–control study
Source: Pediatr Res. 2022 Jul 19;93(4):1096–104. doi: 10.1038/s41390-022-02188-4 (PMC10033398; doi:10.1038/s41390-022-02188-4)
Supplement: Supplementary file 1 — Supplementary Information [file 41390_2022_2188_MOESM1_ESM.pdf]

Supplementary Table 1. Number and types of antibiotic purchases before index date[a] in the matched case-control study of 950 children as compared with antibiotic purchases in the whole Fin-HIT cohort of 11 407 children (born 2000-2005) from birth until 31 December 2018.

**Antibiotic types**	**Number of antibiotic purchases before index date[a] in the matched case-control study**<br><br>**N= 8 604 (%)**	**Number of all antibiotic purchases in the Fin-HIT cohort**<br><br>**N=130 440 (%)**
**Penicillins, N(%)**	3 453 (100) – **40.1%**	52 633 (100) – **40.4%**
Phenoxymethylpenicillin (J01CE02)	431 (12.5)	8 399 (16.0)
Amoxicillin (J01CA04)	3 009 (87.1)	43 657 (82.9)
Flucloxacillin (J01CF05)	0	42 (0.1)
Pivmesillinam (J01CA08)	13 (0.4)	535 (1.0)
**Macrolides, N(%)**	1 949 (100) – **22.7%**	26 318 (100) – **20.2%**
Erythromycin (J01FA01)	2 (0.1)	56 (0.2)
Roxithromycin (J01FA06)	4 (0.2)	188 (0.7)
Clarithromycin (J01FA09)	229 (11.7)	3 737 (14.2)
Azithromycin (J01FA10)	1 714 (87.9)	22 325 (84.8)
Telithromycin (J01FA15)	0	12 (0.0)
**Amoxicillin-clavulanic acid (**J01CR02), **N(%)**	1 295 (100) – **15.1%**	18 262 (100) – **14.0%**
**Cephalosporines, N(%)**	1 202 (100) – **14.0%**	20 993 (100) – **16.1%**
Cephalexin (J01DA01)	1 023 (85.1)	18 713 (89.1)
Cefuroxime (J01DA06)	33 (2.7)	435 (2.1)
Cefaclor (J01DA08)	115 (9.6)	1 500 (7.1)
Cefadroxil (J01DA09)	30 (2.5)	338 (1.6)
Ceftriaxone (J01DA39 and J01DA63)	1 (0.1)	7 (0.0)
**Sulphonamides and trimethoprime, N(%)**	168 (100) – **7.2%**	8 958 (100) – **6.9%**
Trimethoprime (J01EA01)	65 (10.5)	860 (9.6)
Trimethoprime sulphate (J01EE01 and J01EE02)	553 (89.5)	8 098 (90.4)
**Tetracyclins, N(%)**	54 (100) – **0.6%**	2 555 (100) – **2%**
Doxicyclin (J01AA02)	30 (55.6)	1 097 (42.9)
Lymecyclin (J01AA04)	20 (37.0)	1 049 (41.1)
Tetracyclin (J01AA07)	4 (7.4)	409 (16.0)
**Clindamycin** (J01FF01), **N(%)**	23 (100) – **0.3%**	443 (100) – **0.3%**
**Fluoroquinolones**	4 (100) – **0.0%**	220 (100) – **0.2%**
Ciprofloxacin (J01MA01 and J01MA02)	4 (100)	193 (87.7)
Levofloxacin (J01MA02)	0	19 (8.6)
Moxifloxacin (J01MA14)	0	8 (3.6)
**Others** (nitrofurantoin (J01XE01) and metronidazole (J01XD01 and P01AB01), **N(%)**	6 (100) – **0.1%**	58 (100) – **0.0%**

[a] Index date= date of diagnosis for children who developed autoimmune diseases and compatible date for their matching controls.

Supplementary Table 2. Association between types and age of first antibiotic purchase and the onset of autoimmune diseases (DM, AIT, JIA, and IBD).

**First antibiotic purchase**	**AD (N=242)**	**Matched controls (N=708)**	**DM (N=102)**	**Matched DM controls (N=280)**	**AIT (N=68)**	**Matched AIT controls (N=190)**	**JIA (N=54)**	**Matched JIA controls (N=156)**	**IBD (N=27)**	**Matched IBD controls (N=73)**
**Median age, years (IQR)**	1.06 (0.65-1.82)	1.00 (0.65-1.69)	1.09 (0.71-1.84)	1.02 (0.68-1.85)	1.10 (0.74-2.09)	0.97 (0.60-1.68)	0.84 (0.50-1.30)	1.11 (0.65-1.70)	1.15 (0.72-1.87)	0.88 (0.56-1.56)
**Antibiotic type, N(%)**										
No antibiotics	14 (5.8)	34 (4.8)	9 (8.8)	16 (5.7)	2 (2.9)	4 (2.1)	4 (7.4)	11 (7.1)	0	2 (2.7)
Penicillin	133 (55.0)	386 (54.5)	56 (54.9)	157 (56.1)	40 (58.8)	93 (49.0)	27 (50.0)	85 (54.5)	16 (59.3)	45 (61.6)
Cephalosporine	15 (6.2)	58 (8.2)	6 (5.9)	21 (7.5)	4 (5.9)	20 (10.5)	4 (7.4)	13 (8.3)	1 (3.7)	4 (5.5)
Macrolide	51 (21.1)	135 (19.0)	25 (24.5)	57 (20.4)	14 (20.6)	43 (22.6)	11 (20.4)	21 (13.5)	2 (7.4)	12 (16.4)
Amoxicillin-clavulanic acid	25 (10.3)	85 (12.0)	5 (4.9)	23 (8.2)	7 (10.3)	28 (14.7)	6 (11.1)	25 (16.0)	8 (29.6)	9 (12.3)
Sulphonamides and trimethoprim	4 (1.6)	10 (1.4)	1 (1.0)	6 (2.1)	1 (1.5)	1 (0.5)	2 (3.7)	1 (0.6)	0	1 (1.4)
Others	0	1 (0.1)	0	0	0	1 (0.5)	0	0	0	0

AD=autoimmune diseases. Cases=children with ADs (represented with DM (type 1 diabetes mellitus), AIT (autoimmune thyroiditis), JIA (juvenile idiopathic arthritis), and IBD (inflammatory bowel diseases)). Nine children had two diagnoses. IQR=Interquartile Range. The association between types of first antibiotics and the onset of ADs were estimated using conditional logistic regression with penicillin as reference. P>0.05 in every analysis comparing cases and matched controls.

Supplementary Table 3. Additional information for Figure 3. Association between types of antibiotic purchases in different periods and the development of an autoimmune disease (AD), represented by type 1 diabetes (DM), autoimmune thyroiditis (AIT), juvenile idiopathic arthritis (JIA), or inflammatory bowel diseases (IBD)[a].

**Antibiotic types in different periods**	**AD (N=242)**	**DM (N=102)**	**AIT (N=68)**	**JIA (N=54)**	**IBD (N=27)**
	**OR (95%CI)[b]**	**OR (95%CI)**	**OR (95%CI)**	**OR (95%CI)**	**OR (95%CI)**
**Throughout childhood (From birth to index date[c])**					
Penicillin	1.01 (0.96-1.06)	1.05 (0.97-1.14)	0.96 (0.88-1.05)	1.06 (0.94-1.19)	1.00 (0.87-1.14)
Cephalosporine	1.03 (0.95-1.12)	1.04 (0.88-1.22)	0.93 (0.82-1.06)	*1.25 (1.03-1.52)*	1.16 (0.88-1.52)
Macrolide	1.04 (0.90-1.09)	1.04 (0.94-1.16)	0.97 (0.88-1.08)	*1.21 (1.06-1.38)*	1.03 (0.94-1.13)
Amoxicillin-Clavulanic acid	1.05 (0.98-1.13)	1.02 (0.88-1.18)	0.97 (0.85-1.10)	*1.19 (1.02-1.39)*	1.15 (0.98-1.34)
Sulphonamides and trimethoprim	1.08 (1.00-1.16)	1.12 (0.98-1.29)	1.04 (0.94-1.14)	1.31 (0.96-1.78)	1.09 (0.82-1.45)
**Infancy (<age of 1 year)**					
Penicillin	1.45 (0.90-2.32)	0.90 (0.62-1.30)	0.77 (0.49-1.23)	1.06 (0.68-1.65)	0.53 (0.23-1.19)
Cephalosporine	0.94 (0.64-1.38)	0.76 (0.37-1.57)	0.68 (0.30-1.51)	*2.54 (1.01-6.38)*	0.50 (0.08-3.00)
Macrolide	1.00 (0.78-1.28)	0.87 (0.57-1.32)	0.83 (0.53-1.31)	*1.80 (1.08-3.01)*	0.47 (0.14-1.61)
Amoxicillin-Clavulanic acid	1.07 (0.77-1.49)	0.65 (0.30-1.41)	0.65 (0.30-1.38)	*1.93 (1.12-3.32)*	1.17 (0.34-4.03)
Sulphonamides and trimethoprim	1.26 (0.89-1.78)	1.15 (0.70-1.87)	0.65 (0.30-1.38)	1.21 (0.19-7.58)	0.50 (0.01-23.6)
**Toddler phase (From age of 1 up to third birthday)**					
Penicillin	1.06 (0.95-1.19)	1.15 (0.97-1.35)	1.08 (0.85-1.38)	1.02 (0.77-1.35)	1.03 (0.76-1.40)
Cephalosporine	1.11 (0.92-1.33)	1.05 (0.78-1.41)	0.76 (0.51-1.12)	*1.69 (1.15-2.48)*	1.57 (0.81-3.03)
Macrolide	1.09 (0.97-1.21)	1.09 (0.91-1.31)	1.01 (0.84-1.21)	*1.40 (1.09-1.79)*	0.83 (0.58-1.19)
Amoxicillin-Clavulanic acid	*1.18 (1.01-1.37)*	1.22 (0.98-1.54)	0.94 (0.70-1.25)	*1.43 (1.03-2.00)*	1.30 (0.79-2.15)
Sulphonamides and trimethoprim	1.10 (0.94-1.29)	1.10 (0.84-1.44)	1.12 (0.89-1.41)	1.63 (0.77-3.47)	0.91 (0.46-1.77)
**Preschool to adolescence (From age 3 years to index date)**					
Penicillin	1.01 (0.94-1.08)	1.04 (0.92-1.17)	0.94 (0.84-1.06)	1.09 (0.94-1.28)	1.02 (0.86-1.22)
Cephalosporine	1.02 (0.92-1.13)	1.07 (0.87-1.33)	0.97 (0.84-1.12)	1.12 (0.86-1.46)	1.13 (0.84-1.53)
Macrolide	1.04 (0.97-1.12)	1.09 (0.89-1.33)	0.94 (0.70-1.12)	1.19 (0.98-1.45)	1.06 (0.95-1.18)
Amoxicillin-Clavulanic acid	1.03 (0.93-1.13)	0.89 (0.69-1.16)	1.00 (0.84-1.19)	1.12 (0.91-1.38)	1.18 (0.98-1.42)
Sulphonamides and trimethoprim	1.10 (0.98-1.24)	*1.35 (1.03-1.77)*	1.00 (0.84-1.16)	1.26 (0.89-1.78)	1.45 (0.90-2.32)
**Purchases within 2 years before index date**					
Penicillin	0.96 (0.80-1.14)	0.81 (0.61-1.08)	1.03 (0.71-1.50)	1.19 (0.84-1.71)	0.97 (0.58-1.62)
Cephalosporine	1.19 (0.98-1.45)	1.22 (0.94-1.57)	1.15 (0.78-1.71)	1.01 (0.56-1.82)	2.19 (0.90-5.32)
Macrolide	*1.24 (1.01-1.51)*	1.12 (0.83-1.51)	0.87 (0.38-2.03)	1.35 (0.91-2.00)	1.29 (0.81-2.04)
Amoxicillin-Clavulanic acid	1.04 (0.83-1.31)	1.01(0.70-1.48)	0.44 (0.17-1.11)	1.33 (0.92-1.93)	1.23 (0.45-3.39)
Sulphonamides and trimethoprim	1.15 (0.84-1.57)	0.60 (0.22-1.64)	1.19 (0.80-1.76)	1.25 (0.64-2.44)	8.08 (infinity)

[a] Cases=children with ADs (DM, AIT, JIA or IBD). Nine children had two diagnoses

[b] OR=Odds ratio, CI= 95% Confidence Interval. Analyses was performed using conditional logistic regression.

[c] Index date= date of diagnosis for children who developed autoimmune diseases and compatible date for their matching controls.

Supplementary Figure 1

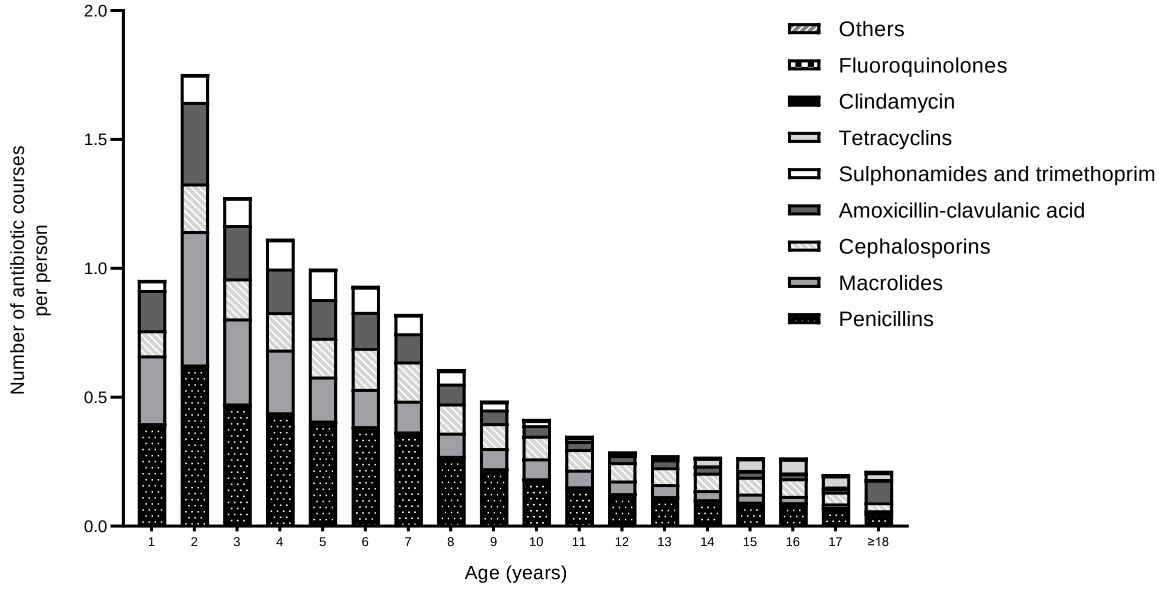

Title: All purchased per person antibiotics in the cohort of 11,407 children born in 2000-2005. Number of tetracyclin, clindamycin, fluoroquinolone, and other antibiotics purchases were low.
